# NO Dioxygenase Activity in Hemoglobins Is Ubiquitous *In Vitro*, but Limited by Reduction *In Vivo*

**DOI:** 10.1371/journal.pone.0002039

**Published:** 2008-04-30

**Authors:** Benoit J. Smagghe, James T. Trent, Mark S. Hargrove

**Affiliations:** Department of Biochemistry, Biophysics, and Molecular Biology, Iowa State University, Ames, Iowa, United States of America; Monash University, Australia

## Abstract

Genomics has produced hundreds of new hemoglobin sequences with examples in nearly every living organism. Structural and biochemical characterizations of many recombinant proteins reveal reactions, like oxygen binding and NO dioxygenation, that appear general to the hemoglobin superfamily regardless of whether they are related to physiological function. Despite considerable attention to “hexacoordinate” hemoglobins, which are found in nearly every plant and animal, no clear physiological role(s) has been assigned to them in any species. One popular and relevant hypothesis for their function is protection against NO. Here we have tested a comprehensive representation of hexacoordinate hemoglobins from plants (rice hemoglobin), animals (neuroglobin and cytoglobin), and bacteria (*Synechocystis* hemoglobin) for their abilities to scavenge NO compared to myoglobin. Our experiments include *in vitro* comparisons of NO dioxygenation, ferric NO binding, NO-induced reduction, NO scavenging with an artificial reduction system, and the ability to substitute for a known NO scavenger (flavohemoglobin) in *E. coli*. We conclude that none of these tests reveal any distinguishing predisposition toward a role in NO scavenging for the hxHbs, but that any hemoglobin could likely serve this role in the presence of a mechanism for heme iron re-reduction. Hence, future research to test the role of Hbs in NO scavenging would benefit more from the identification of cognate reductases than from *in vitro* analysis of NO and O_2_ binding.

## Introduction

Hemoglobins (Hbs) became a focus of study early in the history of biochemistry, due to their color and ready availability in red blood cells and muscle. An unambiguous role facilitating respiration through oxygen transport made red blood cell Hb the first protein for which a physiological function was understood [1]. A clear physiological role in combination with facile structural analysis has made oxygen transport Hbs ideal models for those seeking to learn the biophysical details relating protein structure and function [2], [3]. Recent years however, have seen new Hb discoveries that challenge many paradigms in this field, including that of their predominant function as oxygen transporters. For example, it is clear that red blood cell Hb and Mb also play important roles in NO homeostasis [4]–[7].

Genomics studies have generated hundreds of new globin sequences and revealed this family of proteins as being nearly ubiquitous among living organisms [8]–[10]. Hexacoordinate Hbs (hxHbs) are a structurally unique subset of the Hb superfamily that display reversible coordination of the heme iron ligand-binding site by an intramolecular histidine side chain [11]–[14]. Prominent members of this group include neuroglobin and cytoglobin from humans and other animals [9], [11], [12], [15]–17, the nonsymbiotic plant Hbs (nsHbs) found in all plants [14], and the cyanobacterial protein cyanoglobin [18], [19]. The diverse and prevalent distribution of hxHbs among organisms is often coupled with their high cross-species sequence identity, suggesting proteins with critical physiological roles. Current hypothesis suggest they might provide a common mechanism for protecting cells against hypoxia and oxidative chemistry in plants and animals. Despite numerous publications describing the significant structural and biophysical effort directed at these proteins [11]–[13], [19]–[25], a solid assignment of their physiological role(s) remains elusive [8], [14], [26].

While many enzymes exhibit exquisite efficiency and specificity in the catalysis of their reactions, the innate chemistry of the heme prosthetic group is more “promiscuous” in nature, conferring Hbs with several reactivities of potential *in vivo* significance. These vary from reversible binding of diatomic ligands (including O_2_, CO, NO, and many anions) 2, 27, to peroxidase activity [28], [29], and redox reactions [14]. Studies of such reactions have served to focus significant attention on putative functions of newly discovered Hbs involving nitric oxide (NO) binding and/or scavenging [30]–[34]. Such studies include the measurement of NO reactions with hxHbs in the oxy [33], [35], [36], ferric and ferrous protein forms [37], [38], as well as characterization of peroxynitrite reactions with some ferrous-NO hxHb [38], [39].

**Figure 1:**
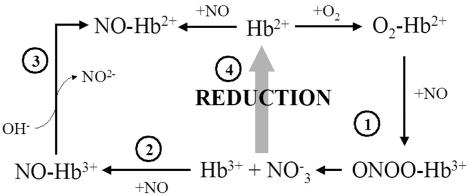
NO reactions with hemoglobins. 1, NO dioxygenase activity; 2, NO binding to the ferric Hbs; 3, NO-induced heme iron reduction; 4, O_2_ nitroxylase activity; 5, reduction reaction of ferric Hbs (4).

Two reaction mechanisms have been proposed for the scavenging of NO by heme proteins. Mb and red blood cell Hb destroy NO using the “NO dioxygenase” (NOD) reaction (Figure 1 #1) in which the oxy-Hb rapidly reacts with NO to form ferric Hb and nitrate [40], [41]. In fact, even when ferrous NO-Hb is reacted with O_2_, the rate limit to heme oxidation is NO dissociation, which is rapidly followed by O_2_ binding to the heme iron and a subsequent NOD reaction (49). Initially it was reported that the bacterial and yeast flavohemoglobins (flavoHbs) use the NOD mechanism in their reactions [42]–[46]. Alternatively, it has been proposed that the flavoHbs use an “O_2_ denitroxylase” mechanism (Figure 1 #4), in which NO binding precedes a reaction between the NO-heme complex and oxygen [47]–[49]. In either case, the resulting oxidized heme must be reduced to start the reaction over (Figure 1, #5). For flavoHbs, rapid reduction is achieved through a flavin-containing reductase domain [50]–[54].

Because of the change in heme oxidation state that accompanies NO reactions with Hbs, reduction reactions have been a recent focus of research [55]–[57]. The kinetics of the reduction reaction is not as critical in NO scavenging by red blood cell Hb or Mb, as these proteins are present in vast stoichiometric excess compared to NO. However, with the possible exception of neuroglobin in the retina [58], hxHbs are present in very low (sub-micromolar) concentrations in the organisms in which they have been identified [14], [26]. Thus, if they were to serve catalytic roles in NO scavenging, efficient mechanisms for reduction would be needed. However, cognate reductases suitable for reduction on flavoHb time scales have yet to be assigned for any hxHb and reduction mechanisms are still unknown. Thus an important question to ask for hxHbs is whether reduction is truly the rate limiting step for catalytic NO scavenging. Ferric Hbs can also bind NO (Figure 1, #2) and, as bound NO can potentially reduce the heme iron (Figure 1, #3) [38], [59], ferric NO reactions and NO-reduction must be evaluated for hxHbs as well.

The purpose of the research reported here is to systematically compare NO reactions with a representative set of hxHbs to Mb, with the underlying goal of testing whether these reactions distinguish any of the hxHbs in their ability to bind or destroy NO. The importance of this comparison centers on the premise that if we are justified in using *in vitro* reactions to assign NO binding functions in hxHbs, we should expect some characteristic to distinguish a hxHb from Mb, which does not catalyze NO destruction absent a reductase. Here we compare NO scavenging *in vitro* and in flavoHb knockout *E. coli* cells and ferric NO reactions with human neuroglobin (Ngb) and cytoglobin (Cgb), rice nsHb (riceHb1), *Synechocystis* hemoglobin (cyanoglobin, *Syn*Hb), and horse heart Mb. Our results demonstrate that all of these oxyHbs can rapidly destroy NO *in vitro* at a rate equal to re-reduction of the ferric Hb, all bind NO in the ferric form, all but ferric *Syn*Hb are slowly reduced by NO, but only the bacterial hxHb can replace flavoHb function *in vivo*.

## Materials and Methods

### Protein production and purification

Human neuroglobin (Ngb, GenBank accession number Q9NPG2) and cytoglobin (Cgb, GenBank accession number Q8WWM9), rice nsHb (riceHb1, GenBank accession number O04986) and *Synechocystis* hemoglobin (*Syn*Hb, GenBank accession number BAA17991) were expressed and purified as described previously [22]. Horse heart myoglobin was commercially obtained (Sigma), dissolved in 0.1 M potassium phosphate (pH 7.0) to generate a 2 mM stock, and desalted over a G-25 column. This procedure yielded a sample giving a single band on SDS PAGE with kinetic and spectral properties identical to those previously published [2]. Prior to experiments, all proteins were oxidized with an excess of potassium ferricyanide, which was removed by passage through a Sephadex G-25 column equilibrated in 0.01M potassium phosphate (pH 7.0). The ferredoxin-NADP reductase gene (GenBank accession number AAC76906) was amplified by PCR from *Escherichia coli*. The oligonucleotide sequences were: 5′CCTGGTGCCGCGCGGCAGCCATATGGCTGATTGGGTAACAGGCAAAG–3′ and 5′-TTGTCGACGGAGCTCGAATTCTTACCAGTAATGCTCCGCTGTC-3′. Amplification was done by 35 cycles of 30s at 95°C, 30s at 55°C and 1 min at 72°C. The resulting fragment was then cloned in the pET28a plasmid (Novagen) between the NdeI and EcoRI restriction sites. The His-Tagged protein was expressed and purified as described earlier [22].

### UV/VIS spectroscopy

The NO-ferric spectra were recorded by mixing a deoxygenated ferric Hb solution (5 µM) with a 2 mM NO solution. The NO-ferrous spectra were recorded by addition of a 2 mM NO solution (1mM final) to a sodium dithionite reduced protein solution, in a cuvette previously sparged with N_2_. To follow the NO-induced reduction of the protein, spectra were collected over a period of 5h after mixing.

First attempts to measure ferric NO binding displayed non-saturated spectrum at low NO concentrations. Therefore, NO binding was measured by equilibrium titration of 5 µM Hb with NO solutions (7 µM to 2 mM). The dissociation equilibrium constants (K_D NO, Fe3+_) for each protein (Table 1) were extracted using the following equation where F_B_ is the fraction of NO-bound protein:
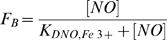
(Equation 1)


**Table 1:** Kinetic and affinity values for *in vitro* reactions with NO and heme iron reduction.

	k_obs, NOD_	K_DNO (Fe3+)_ µM	k_obs,NO (Fe3+)_ mM^−1^s^−1^	k_red,NO_ min^−1^	k_cat_ vs FdR min^−1^	K_m_ vs FdR µM	Observed Hb reduction µM/min^−1^	Observed NO consumption µM/min^−1^
Mb	34 (µM^−1^s^−1^)	262	70	0.03	5	17	2	3.2
RiceHb1	90* (s^−1^)	42	3.5	0.0006	5	20	1.6	2.9
Ngb	360* (s^−1^)	75	2	0.12	6	17	1.9	2.7
Cgb	430* (s^−1^)	17	13	0.05	9	21	2.7	5.3
*Syn*Hb	na	370	0.073	∼0	5	12	2.4	2.2

* : maximal observed rate of NOD

The saturated NO solution (2 mM) was prepared by equilibrating a buffer solution with NO gas that was first passed through a 20% NaOH solution [60]. All spectra were recorded using a Varian Cary 50 spectrophotometer at room temperature. All solutions were made with 0.1 M potassium phosphate buffer pH 7.0.

### NO dioxygenation and scavenging *in vitro*

A multi-port measurement chamber (World Precision Instruments, WPI. Sarasota, Fl) was used to analyze the stoichiometric reaction between oxyferrous hxHb and NO at room temperature. The chamber contained 0.1 M potassium phosphate pH 7.0 and was equilibrated with N_2_. The low oxygen concentration was measured using an oxygen electrode (ISO-OXY-2, WPI). At 0% oxygen, the chamber was closed leaving a small dead space (0.5 cm) at the surface of the solution flushed with N_2_. Then 20 µM NO was added and the signal was followed using a NO electrode (ISO-NOP, WPI). When it reached a maximum, oxyferrous Hb (20 µM final concentration) was added to the solution and the decay of the NO signal was recorded.

Rapid mixing experiments measuring NOD rate constants have been described earlier [40] and were conducted with a BioLogic SFM 400 stopped-flow reactor coupled to a MOS 250 spectrophotometer. Ferric protein was reduced using a system adapted from an earlier study [61] in an Eppendorf sparged with N_2_. After 10 min, each ferrous protein sample was added to a gas tight syringe containing 0.1 M potassium phosphate pH 7.0 (262 µM O_2_). The oxygen bound protein (1 to 5 µM, after mixing) was confirmed by recording the oxy-ferrous spectrum. The NO solutions were generated by mixing a saturated NO solution (2 mM) with a N_2_ equilibrated potassium phosphate buffer (0.1 M, pH 7.0) in gas tight syringes. An anaerobic condition in the NO syringes was maintained using the glucose oxidase-catalse system [35]. Kinetic time courses were collected (20°C) at different NO concentrations (5 to 500 µM) by recording the change in absorbance at a fixed wavelength (413 for Mb, 422 for Ngb/Cgb and 420 for riceHb1). Between five and eight kinetic traces were collected and averaged for each time course.

### Ferric NO binding

Rapid mixing experiments measuring NO binding to the ferric protein were conducted with a BioLogic SFM 400 stopped-flow reactor coupled to a MOS 250 spectrophotometer. Two syringes were used: one containing the protein solution (Fe^3+^, ∼5 µM after mixing) and the other containing an NO solution. Reaction concentrations above 1 mM [NO] were obtained by mixing a larger volume of the saturated NO solution than volume of Hb. Anaerobic condition in the NO syringes were maintained using the glucose oxidase-catalse system [35]. Kinetics time courses were collected (20°C) at different NO concentrations by recording the change in absorbance at a fixed wavelength (Soret peak). At least three kinetic traces were collected and averaged. The minimal NO concentration was at least 2 times higher than the calculated K_D NO, Fe3+_ (Table 1).

### Enzymatic reduction of hxHbs

The reduction system used in the present study has been adapted from one described in detail previously [61]. First a 1 ml cuvette was flushed for 1 min with 1 atm CO. Then 0.1 M potassium phosphate pH 7.0 equilibrated with 1 atm CO was added through a rubber stopper. After addition of 60 µM NADP^+^ (Sigma, St. Louis, MO), 0.7 U/ml glucose-6-phosphate (G6P) dehydrogenase (Roche, Pleasanton, CA), 1 µM ferredoxin-NADP reductase (*E. coli*) and 5 µM Hb, 3mM G6P was added to start the reduction reaction. Absorbance spectra were collected over a period of 30 min at room temperature. The difference in absorbance between ferric and CO-bound protein was plotted versus time to calculate the initial velocity of the reaction.

### Catalytic NO consumption experiments

The multi-port measurement chamber (WPI) containing 0.1 M potassium phosphate pH 7.0 and 3mM G6P, was first equilibrated with a mixture N_2_/O_2_ to reach an O_2_ concentration of 4%, as measured with an oxygen electrode (ISO-OXY-2, WPI). At 4% O_2_, the chamber was closed leaving a small dead space (0.5 cm) at the surface of the solution. Then, 10 µM Hb, 60 µM NADP^+^ (Sigma, St. Louis, MO), 0.7 U/ml glucose-6-phosphate (G6P) dehydrogenase (Roche, Pleasanton, CA), and 1 µM ferredoxin-NADP reductase (*E. coli*) was added to the solution to make the ferrous-oxy complex. After 10 min, 40 µM NO was added to the solution and its removal was measured using the NO electrode (ISO-NOP, WPI) at room temperature. The NO consumption rate (Table 1) was calculated from the initial velocity just after NO addition.

### NO scavenging in flavoHb knockout *E. coli*

*E.coli* strains AB1157 [42] and AG1000 ((AB1157*Φ* (*hmp-lacZ*)262; *Cm^r^*)) were generously provided by Dr Paul Gardner (Cincinnati Children's Hospital Medical Center). All Hbs were cloned into the pANX plasmid between the NdeI and HindIII restriction site except sperm whale myoglobin, which was cloned between the NdeI and BamHI restriction sites. The pANX plasmid is derived from pUC19 and contains the promoter region of the *hmp* gene of *E. coli* [62]. The expression of a gene cloned next to it (via NdeI), will be driven by that promoter and should be comparable to the natural *hmp* gene expression. The generated plasmids were transformed into the *hmp* deficient strain AG1000. As a control, “virgin” pANX and pANX-hmp (provided by Dr Paul Gardner) were also transformed into AG1000.

A test tube containing 5 ml LB medium was inoculated with 1% of an overnight culture grown aerobically. No selection was used for the AB1157 strain. For the AG1000 strain, 27 g/ml chloramphenicol was added to the medium, and for AG1000 containing the different plasmids, 50 µg/ml carbenicillin was also added. After inoculation of the test tubes (∼0.02 OD_600_), 3 mM GSNO was added and the cultures were grown aerobically at 37°C with constant agitation. The concentration of GSNO used in these experiments was determined by the minimum level necessary to reveal a clear hmp- phenotype in our reactions. (and is comparable to those used by others in NO challenge experiments). After 14 hours the OD_600_ was measured. As a control, cells were grown without GSNO and in the presence of 3 mM inactivated GSNO (as described in the Supplemental Figure S1.) Untreated cells were also used for the measurement of NO consumption. The results shown are an average of three independent experiments, repeated once.

GSNO was generated by mixing a stoichiometric amount of reduced gluthathione (Sigma) and acidified sodium nitrite for 10 min in the dark [63]. Then the pH was adjusted to 7.4 with 4N sodium hydroxide. The concentration of GSNO was determined using ε_334_ = 767 M^−1^cm^−1^ [64]. As control, to test if the effect observed is due to NO alone or to other by-products produced during its synthesis, we destroyed the NO component of our GSNO by photolysis [65]. To do this, the GSNO solution (∼0.2 M) was placed in a 4 ml cuvette and photolyzed using a YAG laser (532 nm). After 45 min all GSNO was inactivated, and NO released during that process was neutralized by pure oxygen during a 15 min exposure. These experiments are provided in Supplemental Figure S1.

### Measurement of hemoglobin expression levels *in vivo*

Saturated cultures, inoculated from an overnight culture, were used to measure the expression level for each Hb. For *Syn*Hb, cells treated overnight with 3 mM GSNO were also used. Cells were harvested by centrifugation at 6000 rpm for 6 min. The cell pellet was then resuspended in 0.1 M potassium phosphate pH 7.0 and sonicated (3×30s with ∼5 min interval). After centrifugation to remove the cell debris (20000 rpm for 30 min), the supernatant (50 mg/ml total protein) was used to record CO+sodium dithionite–reduced sodium dithionite difference spectra. The difference spectrum of each protein was corrected by the difference spectrum measured with the supernatant of the strain transformed with pANX alone. The results shown are an average of two independent experiments.

### Measurement of NO consumption *in vivo*

The multi-port measurement chamber (WPI) containing air equilibrated 0.1 M phosphate pH 7.0 was used for the aerobic NO consumption, at room temperature, of the different *E. coli* strains. NO (2 µM) was added to the chamber, and when the signal reached a maximum (as measured with the NO electrode (ISO-NOP, WPI)) cells were added (1×10^7^ cells/ml) from an overnight culture. For *Syn*Hb, cells treated overnight with 3 mM GSNO were also used. The rate of consumption was calculated as the time required to consume 1 µM (half the signal) of NO. All rates were corrected for the rate of consumption by the strain transformed with pANX alone. The results shown are an average of six independent experiments, repeated once.

## Results

### NO dioxygenation and non-catalytic NO consumption

**Figure 2:**
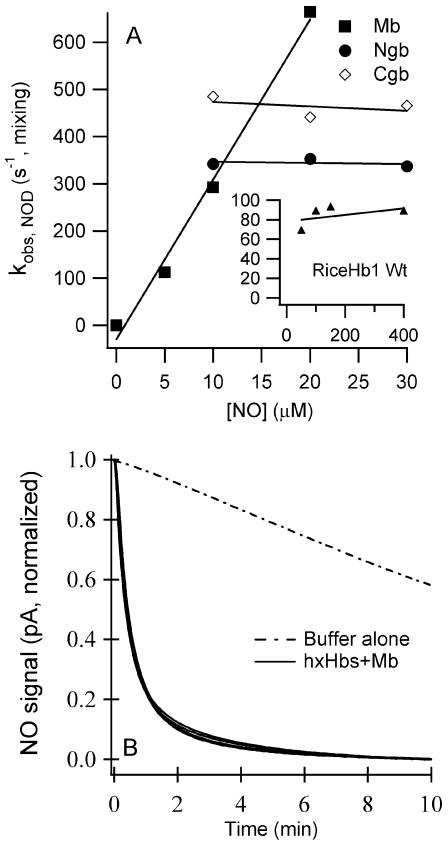
NO dioxygenation and stoichiometric NO consumption. Plots of k_obs,NOD_ vs [NO] for the NOD reaction following rapid mixing.

The rate constant for NOD by Mb has been reported previously [40]. It is a very rapid (nearly diffusion limited), bimolecular reaction between NO and the oxy-Hb complex. While peroxynitrite is considered to be an intermediate in this reaction (Figure 1, #1) [66], its dissociation rate is not limited in Mb over the range of NO concentrations amenable to stopped flow kinetics [40]. All of the oxy-hxHbs react with NO, and NOD by Ngb and a plant nsHb has been reported previously [35], [67], [68]. In the case of mouse Ngb, the reaction displayed similar kinetics at the two [NO] tested [35]. The interpretation of this observation was that dissociation of peroxynitrite is rate limiting. Each hxHb investigated here, over a range of NO concentrations, showed saturating kinetics as [NO] increased, but the limiting observed rate constant varies between proteins (Figure 2A). Ngb and Cgb are similar (k_obs, NOD_ = 360 and 430 s^−1^, respectively), but riceHb1 is slower (k_obs, NOD_ = 90 s^−1^) (Table 1). Unfortunately, we were not able to calculate the rate constant for *Syn*Hb as the change in absorbance was very small, resulting in very noisy time courses.

### Ferric NO binding and NO-induced reduction

**Figure 3:**
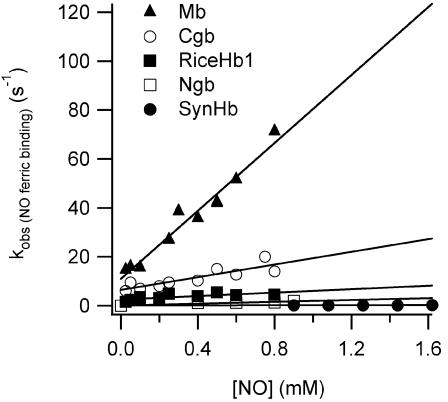
NO binding to ferric Mb and hxHbs. Plots of k_obs_ versus [NO] for Mb and each hxHb. Time courses giving rise to these values were measured at different [NO] ranging from 50 to 1700 µM (after mixing) and were fitted to a single exponential to extract the observed rate constants (k_obs_). A linear fit of these data provides the observed ferric NO binding association rate constant (k_obs, NO(Fe3+)_).

Human Ngb is the only ferric hxHb for which reactions with NO have been published [38]. Binding was very slow, and produced ferrous NO-Ngb. Figure 3 shows the [NO] dependence of the observed rate constant following mixing with each ferric hxHb and Mb. Mb binds NO in a bimolecular fashion, with an observed rate constant equal to 70 mM^−1^ s^−1^. In the case of each hxHb, time courses for binding also display a linear dependence on [NO], but the observed bimolecular rate constants are much smaller (k_obs,NO(Fe3+)_, Table 1).

**Figure 4:**
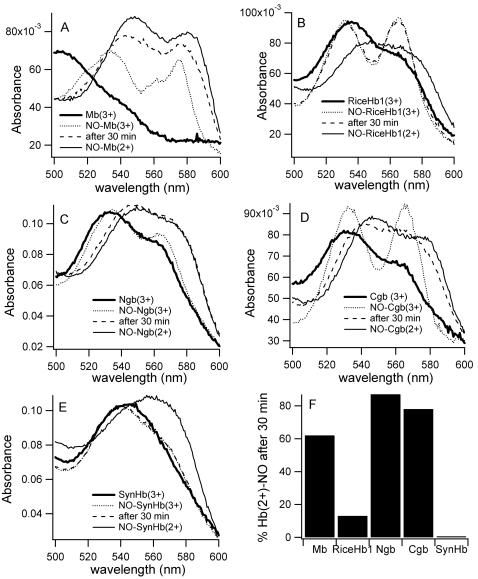
Absorbance spectra associated with NO-induced reduction. Panels (A–E) show the Hb(3+) (thick solid line), Hb(3+)-NO (dotted line), Hb(2+)-NO (thin dotted line) absorbance spectra, and the spectrum of the sample 30 minutes after mixing NO with ferric protein (dashed line). Ferric and ferrous oxidation states are indicated by 3+ and 2+, respectively. F. Percentage of Hb(2+)-NO after 30 minutes of reduction.

NO reduction of ferric Hbs has been reported extensively for Mb [59], [69], and as described above for Ngb [38]. Following the NO binding reactions in Figure 3 (particularly those at higher [NO]), relatively slow reduction of some of the ferric Hbs by NO was observed (Figure 4A–E). The degree to which this reaction can regenerate ferrous Hb was gauged by calculating the fraction of Hb(2+)-NO present after 30 min (Figure 4F). Only Mb, Ngb, and Cgb showed significant reduction over this time period. Time courses for this reaction yielded rate constants (k_red,NO_) of 0.03, 0.12 and 0.05 min^−1^ for Mb, Ngb and Cgb, respectively (Table 1). For riceHb1, the rate constant was estimated to be 0.006 min^−1^ (following a 5h time course). Ferric *Syn*Hb showed no significant reduction by NO.

### Catalytic NO scavenging

**Figure 5:**
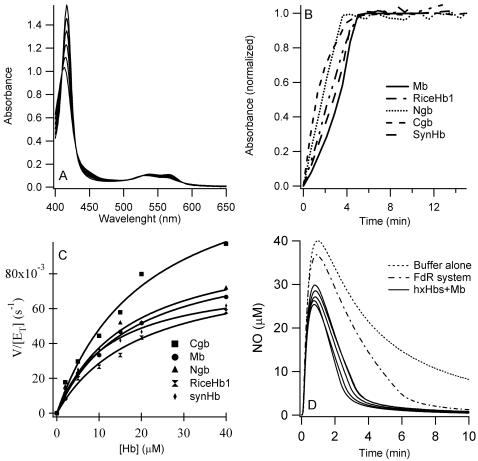
Enzymatic reduction by ferredoxin-NADP reductase and catalytic NO consumption . A. Absorption spectra associated with the reduction of Ngb (10 µM) by 1 µM ferredoxin-NADP reductase in the presence of CO (1 mM). B. Change in absorbance at the Soret peak (CO-bound) associated with the reduction is plotted vs time for each Hb (10 µM). The data are normalized to the absorbance change expected for complete reduction of the Hb in question. C. Enzymatic reduction of Mb and hxHbs by ferredoxin-NADP reductase. V/[E_T_] calculated at different Hb concentration is plotted vs [Hb]. The fit to the Michaelis-Menten equation gives Km and Vmax for reduction of each protein. D. Consumption of 40 µM NO by 10 µM oxyHb as measured by an NO electrode, in the presence of ferredoxin-NADP reductase (1 µM). For each protein, after addition of NO, the signal drops by ∼10 µM corresponding to stoichiometric NOD. [NO] then decreases linearly indicating catalytic NO destruction. Rates of consumption were calculated from the linear phase of catalytic NO removal.

We have demonstrated that each oxy-hxHb has the ability to carry out the NOD reaction with varying degrees of efficiency (Figure 2 and Table 1). However, to scavenge NO catalytically, re-reduction of the resulting ferric heme iron must occur (Figure 1, #5). In Figure 5, the contribution of heme iron reduction to NO scavenging is investigated by using an artificial reductase system (ferredoxin-NADP reductase (FdR) from *E. coli*) that reacts with similar effectiveness with each hxHb under investigation. Figure 5A provides an example of the spectral changes associated with reduction of ferric Ngb by this system and in the presence of CO (which serves to trap the reduced Hb). Figure 5B shows time courses for reduction of each hxHb (10 µM) by 1 µM FdR, and the effect of substrate concentration (Hb) on reduction velocity is shown in Figure 5C. The plots have enough curvature to allow calculation of k_cat_ and K_M_ for each protein (Table 1). K_M_ and k_cat_ are similar for each Hb, with none deviating by more that 2-fold from the others.

Figure 5D demonstrates catalytic NO scavenging in the FdR/Hb system described above. With no Hb present, NO is degraded to 20 µM in ∼5 minutes, and the NO signal reaches the initial baseline value over a period of about 30 minutes. The presence of 10 µM oxy-Hbs accelerates NO removal in a bimodal manner. First, 25% of the signal (10 µM of the NO) is lost rapidly due to stoichiometric NOD. The remaining signal decays linearly back to the background level more rapidly than in the absence of Hb. This rate of NO destruction is attributed to NOD following ferric Hb re-reduction and oxygen binding. In fact, the velocities of NO consumption are nearly identical to the reduction velocities under these experimental conditions (last two columns, Table 1). Therefore, the ability of each hxHb and Mb to catalytically scavenge NO is directly related to the rate of re-reduction of the heme iron following NOD.

### NO scavenging by hxHbs *in vivo*

**Figure 6:**
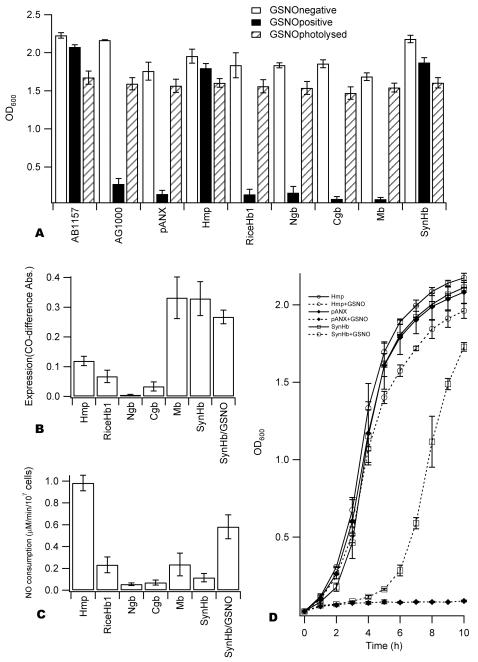
Protection of the *E. coli hmp* mutant from NO by Mb and hxHbs. A. The growth (OD_600_) of wild type (AB1157) and the *hmp* mutant (AB1000) *E coli* strains carrying Mb and hxHbs under control of the flavoHb promoter were measured after 14h in the absence and presence of 3 mM GSNO. Photolyzed GSNO was also used as control. Only flavoHb (*hmp*) and *Syn*Hb are able to rescue the phenotype observed for the wild type strain. The data are an average of the duplication of at least three independent experiments. B. Hemoglobin expression levels driven by the *hmp* promoter. The values reported are the averaged maximum absorbance of the CO-difference spectra peak. Data are an average of two independent experiments. C. NO consumption by different *E. coli* strains. Compared to flavoHb, the consumption by other Hbs is significantly slower. Data are an average of two replications of six independent experiments. D. Growth curves of AG1000 strains transformed with pANX and expressing flavoHb and *Syn*Hb (average of two independent experiments). In the absence of GSNO, no differences are observed between strains. In the presence of GSNO, there is no growth in the strain transformed with pANX alone. With flavoHb, the growth is similar to the one without GSNO. In presence of *Syn*Hb, growth occurs but is significantly retarded.

The only Hbs that are known to be NO scavengers are the bacterial and yeast flavoHbs [42], [70]–[73], and the *E. coli hmp* (flavoHb) null mutant presents a clear NO-sensitive phenotype under aerobic conditions [42], [74]. The experiments in Figure 6 were designed to test the ability of Mb and hxHbs to substitute for flavoHb in *hmp* knockout cells under similar conditions. The foreign Hbs were introduced into the *hmp* mutant cells (AG1000) on a plasmid (pANX) containing the *hmp* promoter, and cell growth was measured in the presence and absence of NO donated by GSNO. GSNO is a NO-releasing agent with properties comparable to spermine/NO or SNAP [75], and was chosen as an NO donor because of its documented ability to up-regulate expression of *hmp* [76]–[78] and therefore expression of the Hbs under the control of the *hmp* promoter on the pANX plasmid. As a precaution, to ensure that the effects we observed were due to NO and not a byproduct of GSNO, “NO depleted” GSNO was also used as a negative control (this procedure is described in the Materials and Methods section and Supplemental Figure S1).

Figure 6A presents OD_600_ values for uniformly-grown *E. coli* cultures after 14 hours [79] showing a comparison of cultures grown in the absence of GSNO, presence of 3 mM GSNO, or the presence of 3 mM GSNO depleted of NO by photolysis. As expected, the wild type strain (AB1157) and the *hmp* mutant strain (AG1000) expressing flavoHb on the pANX plasmid are able to grow in the presence of GSNO, but the AG1000 strain alone and that carrying the empty pANX plasmid are impaired. However, no protection was observed with the AG1000 strains expressing Mb or other hxHbs except the one expressing *Syn*Hb. In the case of *Syn*Hb, cell growth over this time period was indistinguishable from AG1000 expressing flavoHb. The different strains were not sensitive to inactivated GSNO, indicating that the effects observed in the presence of GSNO are only due to the release of NO and not to other compounds present in the GSNO solution.

To investigate whether the results in Figure 6A are attributable to variation in hxHb expression levels in the different strains, expression was measured independently by recording the CO-difference spectrum of each [80]. The level of expression is correlated with the maximum absorbance of the difference between the cell extract (supernatant) with and without addition of CO and sodium dithionite. Figure 6B and Table 2 report these values. From these data it is evident that expression levels vary significantly, with Ngb being poorly expressed and Mb/*Syn*Hb being expressed at the highest concentrations. Compared to flavoHb, RiceHb1 and Cgb are expressed 2 to 4 times less, and Mb and *Syn*Hb almost 3 times more.

**Table 2:** *In vivo* expression and NO consumption

	Relative Expression Averaged maximum absorbance (CO difference spectrum peak)	NO consumption (µM/min/10^7^cells)
Hmp	0.12	0.98
Mb	0.33	0.23
RiceHb1	0.07	0.23
Ngb	0.005	0.055
Cgb	0.03	0.07
*Syn*Hb	0.33/0.28*	0.11/0.58*

* : value after overnight treatment with 3 mM GSNO

NO consumption by these cultures was also measured directly to ensure that it correlates with cell viability. Figure 6C shows rates of NO consumption by strains containing each Hb on the pANX plasmid. Rates of consumption by the hxHbs are at least 5 times slower than flavoHb, even for Mb and *Syn*Hb, which are expressed at the highest levels (Table 2). It was surprising that cultures expressing *Syn*Hb did not consume NO, as they were viable in the growth experiments presented in Figure 6A, suggesting that *Syn*Hb in the pANX system can protect against NO without consuming it from the media.

This discrepancy was investigated by monitoring cell growth as a function of time (Figure 6D). In the absence of GSNO, all cultures grew at about the same rate. In the presence of 3 mM GSNO, the cultures expressing Hmp were unaffected, and all others (with the exception of *Syn*Hb) did not grow. Growth of the *Syn*Hb/pANX culture was retarded, but recovered after ∼6 hours to eventually yield the OD_600_ values in Figure 6A that are indistinguishable from those of the Hmp strains. Furthermore, the GSNO treated *Syn*Hb/pANX culture (14h) was capable of more efficient NO consumption (Figure 6C), but did not show increased concentration of *Syn*Hb (Figure 6B).

## Discussion

### Ferric hxHbs do not efficiently catalyze NO destruction

It has been proposed that hxHbs might destroy NO through a mechanism that includes binding to the ferric heme iron [38]. In the absence of specific reduction mechanisms, this route is compelling due to the potential for NO to reduce the heme, which could then bind oxygen and go through one cycle of NO-dioxygenase activity to reform the starting ferric Hb complex. One complete cycle would scavenge two molecules of NO using two different chemical mechanisms. The results presented in Figures 3 and 4, and Table 1 do not support this hypothesis for any of the hxHbs investigated here. There are at least two kinetic hurdles for this mechanism; both ferric NO binding and NO-induced reduction must be fast. We have demonstrated that binding of NO to ferric hxHbs is significantly slower than to Mb. This is probably due to intramolecular His binding to the ligand binding site, which is enhanced in the ferric oxidation state [81]. The linear dependence of the reaction with [NO] combined with small observed second-order rate constants (k_obsNO, Fe3+_ in Table 1) is indicative of a bimolecular association rate constant for binding to the pentacoordinate complex that is much slower than the rate constants for His binding and dissociation [22]. The combination of small values of k_obsNO, Fe3+_ and low [NO] *in vivo* would result in very slow NO association relative to flavoHbs under the same conditions [44].

An additional factor diminishing the likelihood of the ferric NO-binding mechanism is the slow rate of reduction of the Hb(3+)-NO complex. Although this reaction clearly varied between the hxHbs tested, none exhibited rates capable of rapid NO destruction even when [NO] is sufficient to saturate the ferric complexes. The fastest reduction was observed in Ngb, where a reaction half-life of ∼6 minutes requires ∼1 mM NO to achieve. This does not support NO reduction of ferric hxHbs as a plausible mechanism for NO scavenging *in vivo* where [NO] rarely exceed ∼200 nM [82], [83]. However, it could be sufficient to generate ferrous NO-Ngb for scavenging of peroxynitrite, preventing its deleterious reaction with CO_2_ [38], or for O_2_-nitroxylase mediated NO scavenging under microaerobic conditions [47].

### HxHbs as NO dioxygenases

In this comparative study of Hb NOD, we find no indication that hxHbs are more efficient in this function than Mb. Instead, we observe a limiting reaction in hxHbs that is probably peroxynitrite dissociation [35] (Figure 2). Hence, Mb would perform better in a NO scavenging role utilizing this reaction mechanism. However, we have also demonstrated that the limiting factor in NO scavenging for all Hbs examined is the re-reduction following NOD (Figure 5D, Table 1). Studies showing that monohydroascorbate reductase increases the NOD activity of barley nsHb [84], and that the isolated Hb domain from *E. coli* flavoHb is insufficient to protect cells during NO challenge [80], are in agreement with our results. Thus assignment of NO dioxygenase activity as a physiological function requires the design of experiments that address reduction mechanisms.

The *E. coli* flavoHb reductase domain and the cognate reductase identified for *Vitreoscilla* Hb satisfy this requirement in work attributing physiological relevance to their NOD activity [50], [85]. However, in the cases of most non-oxygen transport Hbs (including those investigated here), such reductases have not been identified. For example, a “nitric oxide activated deoxygenase” function has been attributed to *Ascaris* Hb based on *in vitro* experiments using NADPH at pH 6.0 to achieve reduction [36]. In this case, these reaction conditions are known to reduce Hbs nonspecifically [86], and would likely endow several Hbs including Mb with *in vitro* NO scavenging activity. Thus, as a general phenomenon, *in vitro* scavenging of NO and O_2_ by Hbs in the presence of a reduction system may give little insight into true physiological function.

The ability of *Syn*Hb to substitute for flavoHb in *E. coli* during NO challenge is the only characteristic that distinguishes any of the hxHbs from the others. The lag in growth following GSNO treatment suggests that an endogenous reductase with activity toward *Syn*Hb is expressed in response to this challenge. The question of whether this reductase has a natural role in NO detoxification in *E. coli*, or whether a homologous reductase is present with similar activity in *Synechocystis*, is currently unanswered. Future studies identifying such a reductase would be taken as support for a NOD function for *Syn*Hb but, more importantly, this observation demonstrates the potential use of *E. coli* AG1000 as a background for reductase screening in co-expression experiments. Co-expression of a reductase (or library containing a reductase) along with a Hb in AG1000 could identify Hb/reductase pairs that are capable of replacing FlavoHb, thus providing targets for identification of cognate reductases for Hbs suspected of providing NOD activity in their native hosts.

In conclusion, the present study has evaluated the capacity of hxHbs to serve as NOD enzymes by testing their abilities to react with NO in the oxyferrous and ferric states, to scavenge NO in an artificial reduction system where the rate of reduction is controlled experimentally, and to replace flavoHb *in vivo* under aerobic conditions. Our results demonstrate that these Hbs have a common ability to react rapidly with NO in the oxyferrous state, and that they will subsequently scavenge NO at a rate limited by re-reduction. It is also clear that reaction of the ferric hxHbs with NO are probably not of physiological significance. These results are not contradictory to a role in NO scavenging, but neither do they preferentially support this hypothesis for any particular hxHb. Instead, they serve to focus research in this area on identification of cognate reductases for each Hb within their natural environments.

## Supporting Information

Figure S1 Photolysis inactivation of GSNO
(0.84 MB TIF)
